# Cultural competency in dietetic diabetes care—A qualitative study of the dietician’s perspective

**DOI:** 10.1111/hex.13019

**Published:** 2020-02-11

**Authors:** Mirjam Jager, Andrea den Boeft, Susanne Leij‐Halfwerk, Rob van der Sande, Maria van den Muijsenbergh

**Affiliations:** ^1^ Nutrition and Dietetics HAN University of Applied Sciences Nijmegen The Netherlands; ^2^ Primary and Community Care HAN University of Applied Sciences Nijmegen The Netherlands; ^3^ Department of Primary and Community Care Radboud University Medical Centre Nijmegen The Netherlands; ^4^ Internal Medicine and Dermatology Department of Dietetics University Medical Centre Utrecht Utrecht The Netherlands; ^5^ Pharos, National Centre of Expertise on Health Disparities Utrecht The Netherlands

**Keywords:** cultural competence, diabetes mellitus, dietetic care, dieticians, ethnic minorities, migrants

## Abstract

**Introduction:**

Diabetes type 2 is more prevalent in ethnic minorities in the Netherlands, and outcomes of health care in general are worse compared to other Dutch patients. The purpose of this study is to explore the experiences of dieticians and the knowledge, skills and attitudes they consider to be important for effective dietetic care in migrant patients.

**Methods:**

Semi‐structured interviews were held with 12 dieticians, of various ages, ethnic backgrounds and experience. The interview guide was based on Seeleman's cultural competence model and the Dutch dietetic consultation model. Interviews were transcribed, coded and thematically analysed, revealing 7 main themes.

**Results:**

Dieticians were uncertain whether their care fulfilled their migrant patients’ needs. They experienced language differences as a major barrier for retrieving information and tailoring advice to the patient's needs. Furthermore, dieticians feel they lack cultural knowledge. An open and respectful attitude was considered important for effective care. The communication barrier hindered building a trusting relationship; however, few dieticians mentioned a need for communication training. They expressed a need for cultural competence training, specifically to acquire cultural knowledge.

**Conclusion:**

Dieticians struggle with providing dietetic care for migrant diabetes patients due to communication barriers and difficulty in building a trusting relationship. They are conscious of their lack of cultural knowledge, and acknowledge the need for an open and respectful attitude and essential communication skills in order to collect and convey information. They seem unaware of the impact of low (health) literacy. Cultural competence training is needed for effective dietetic care for migrants.

## 1 INTRODUCTION

In the Netherlands, at least 22% of the population has an ethnic minority background, half of whom are migrants.^1^ The largest groups originate from Turkey, Morocco, Surinam and the Dutch Antilles.^1^Type 2 diabetes mellitus is highly prevalent within Europe, and two to four times more prevalent in ethnic minority populations in this region.^2^ Additionally, amongst ethnic minorities, higher blood glucose and lipid levels are observed and severe complications occur more often^3^ compared to native Dutch diabetic patients.^4^ These health disparities relate to patients’ poor health literacy^5^, ^6^ and lower self‐efficacy^7^ resulting in poor self‐management. Unhealthy dietary patterns, for example consumption of high amounts of sugar‐rich beverages as well as small amounts of whole‐grain products,^8^ and low physical activity^7^, ^9^ may be rooted in cultural habits with an emphasis on elaborate cooking, joint meals, and health beliefs.^10^, ^11^, ^12^, ^13^ Language differences and a lack of cultural competences as well as prejudices of health‐care professionals interfere with effective communication and care.^14^, ^15^, ^16^ Moreover, trained professional interpreters are hardly used due to the lack of reimbursement within the Netherlands.

Diabetes care in the Netherlands is multi‐disciplinary, concentrated in primary health care. Dutch multi‐disciplinary guidelines^17^ instruct dieticians to focus on promoting self‐management, adherence to dietary advice and engaging in regular exercise.

Previous studies revealed that dieticians’ counselling does not always fit the needs of migrant patients.^10^ Migrants experience difficulties within their community when adhering to dietary advice 10, 11, 14 and often expect a more rigorous, directive approach of dietetic advice, based on medical tests.^10^

To be able to cope well with the needs of migrants and to provide a good quality of care, dieticians should obtain the necessary knowledge, attitude and skills that are defined by Seeleman^5^ as “cultural competence”: *knowledge* about epidemiology of diseases and differential treatment effects in various ethnic groups, *awareness* of how culture shapes individual behaviour, social contexts and one's own prejudices and *skills* to transfer information in a way the patient can understand, to know when external help with communication is needed and to adapt to new situations creatively. Embedding cultural competence in health‐care systems enables systems to provide appropriate care to patients with diverse values, beliefs and behaviours, and it would meet patients’ social, cultural and linguistic needs.^12^ This improves access and equity for all groups in the population, consumer “health literacy,” communication and mutual understanding and it reduces delays in seeking health care and treatment.^13^

Previous work on cultural competency in dietetic care focused on the views and experiences of migrant diabetes patients.^10^ However, little is known about the experiences of dieticians caring for migrant patients nor which knowledge, skills and attitudes they consider important. Therefore, the research question of this explorative study was what are the experiences of dieticians with dietetic care in migrant patients and which knowledge, skills and attitudes do they consider important for effective dietetic care in migrant patients.

## 2 METHODS

### 2.1 Design

The study has an explorative, qualitative design using semi‐structured in‐depth interviews with dieticians involved in diabetes care. The study is part of a research project on the development of a course in culturally sensitive dieticians' care. In this project, migrant patients, migrant students and dieticians are involved. The study was approved by the Medical Ethical Committee of the Central Committee on Research involving Human Subjects region Arnhem and Nijmegen (reference 2014/210).

### 2.2 Study population

A purposive sampling strategy was followed, aiming at a maximum variation amongst dieticians regarding age, ethnic background, experience in dietetic care in general and in caring for migrants. The practice registry of the Dutch Dietetic Association for primary care dietetic practices was screened in areas with a high density of migrant residents. Dieticians were approached by e‐mail explaining the purpose and procedure of the study and were asked for participation. After signing informed consent, an interview was planned. Recruitment continued until theoretical data saturation was reached.^18^ Participating dieticians were offered a free training in cultural competence.

### 2.3 Data collection

The interview guide was developed by the research team based on Seeleman's cultural competence model^5^ and the Dutch dietetic consultation model.^19^ It addressed topics such as the dieticians’ experiences with migrant diabetes patients, the barriers and facilitators for good dietetic care, the knowledge, skills and attitudes that the dieticians considered important, and training needs. The preliminary interview guide was tested in a pilot interview for comprehensibility. The length of the interviews was between 45 and 90 minutes. Interviews were conducted by MJ (dietician) at the practice location of the dietician. All interviews were audiotaped and transcribed verbatim.

### 2.4 Data processing and analysis

The interviews were anonymized and entered in the computer software program Atlas.ti 8. A framework approach based on Seeleman's cultural competence model^5^ and the Dutch dietetic consultation model^19^ was used to analyse the transcripts. The researchers (MJ and AdB) familiarized themselves with the data by reading, re‐reading and summarizing the interview transcripts. Then a coding scheme based on the framework was developed. Additionally, other codes that emerged from the data were added for more specific aspects. To ensure reliability, both MJ and AdB independently coded and analysed the first three interview transcripts. Differences were discussed until a consensus was reached.

Finally, in an iterative process, related themes were identified to structure the results section. The citations were translated from Dutch into English by MJ.

## 3 RESULTS

Twelve dieticians, aged 22 to 61 years, all female, participated in the study. Ten were of Dutch, one was of Bulgarian origin, and one was of Turkish origin (see Table 1). All participants were professionally trained in the Netherlands. Their dietetic working experience ranged from 5 months to 38 years. Their experience working with migrants varied from only a few patients in total to over 10 years of experience working with many migrant patients.

**Table 1:** Characteristics of participating dieticians (n = 12)

Participant code^a^	Ethnicity	Gender	Working experience (years)	Experience working with migrants (yes/no)	Work setting
D1	Dutch	Female	>20	Yes	Individual practice
D2	Dutch	Female	<5	Yes	Group practice
D3	Bulgarian	Female	<5	Yes	Individual practice
D4	Dutch	Female	10‐20	Yes	Group practice
D5	Dutch	Female	<5	Yes	Group practice
D6	Dutch	Female	10‐20	Yes	Group practice
D7	Dutch	Female	>20	Yes	Group practice
D8	Dutch	Female	10‐20	Yes	Group practice
D9	Dutch	Female	10‐20	Yes	Group practice
D10	Dutch	Female	10‐20	Yes	Group practice
D11	Turkish	Female	<5	Yes	Individual practice
D12	Dutch	Female	<5	Yes	Group practice

a Code based on the order of the interviews.

### 3.1 General experience: working with migrants is difficult

All dieticians from Dutch origin regarded a consultation with a migrant patient as challenging or even difficult. Many of them felt insecure or felt that their advice was inadequate. All dieticians strove for good care and were very willing to help their patients to the best of their ability. However, communication problems and insecurity with migrant patients made some dieticians feel uncomfortable with the consultation and left them feeling unsatisfied.

D5: ‘With mister X (..) it was the most difficult conversation I've had in this entire period. That was solely due to the language problem and the fact that he did not like being counselled. But it was also nice, because he was a very nice man. (…) I thought it was very frustrating that I could not quite figure out what he was doing wrong. I could not reach him, so I could do very little. That is very frustrating’.

D7: ‘And with these groups it does not always work as well as when you understand each other's language well. I struggle with that. (…) It is a very unsatisfactory feeling that one can't improve that. A kind of powerlessness in a way.

The two dieticians from non‐Dutch origin seemed less insecure and regarded the difficulties more as an exciting challenge.

As a consequence of the difficulties they experienced, some dieticians expected their dietetic counselling in migrant diabetes patients to be less effective.

D4: ‘We don't have more time for one patient than for another. (…) That automatically means that for a migrant, the goals may be lower than for a Dutch person that's highly educated. They might cost less time. And then one may set very different goals. That's also the reason that she (Mrs X) should have a blood sugar below 10 and with a Dutch person I would have made it below 8’.

D10: ‘I also notice that with Dutch people I go much deeper into “Why do you eat? What does food mean to you? And what do you want from me? What can you expect?” And I feel that one can't really do that part with this group, because it takes so much time’.

There were several dieticians who felt that the compliance to dietetic advice was low in their migrant patients. Additionally, dieticians mentioned examples which they interpreted as a lack of motivation, such as patients missing appointments or food diaries that were not returned.

D3: ‘Filling in a food diary, people just don't do it’.

D6: ‘I find the compliance very meagre, with non‐western patients. Follow‐up appointments are forgotten or they are just not losing weight or not showing up. They end up having done nothing with the advice, while you made an entire Turkish‐Dutch daily menu’.

D2: ‘Not all of course, but I do notice, that many, it comes across like that to me, that it doesn't interest them much’.

Helping patients to make long‐term behavioural change was seen as challenging in many patients, irrespective of their cultural background, but to really understand the motivation and the behavioural patterns of their migrant patients was found particularly difficult.

### 3.2 Skills: achieving shared decision‐making

Many dieticians were trained in shared decision‐making and used motivational interviewing techniques to help their patients change their lifestyle. However, they mentioned that these techniques did not always seem to fit to the expectations of migrant patients. Dieticians said that migrants expect them to take on a more directive role described as ‘telling patients what to eat and what not to eat’, or to give patients a list with daily menus. Although dieticians were willing to take on this role, they often felt uncomfortable with this approach.

D4: ‘I try to use motivational interviewing and let people think for themselves what they want to change. On the other hand I notice that patients want you to take on a doctor's attitude and say “I’m writing you a prescription, and this is what you have to do.” I’m in doubt. Personally I prefer working together and seeing what can be changed. If someone says “I want a list, what should I do. You tell me the things I have to do,” then I sometimes do that’.

On the other hand, the Turkish dietician preferred a directive role. She felt that asking patients about their views on diet adaptation might be perceived as unprofessional.

D11: ‘And if you use motivational interviewing, (…) they might get the feeling that you don't know that much’.

### 3.3 Skills: communication in case of a language barrier

Language differences between dietician and patient were experienced as the major barrier to good care. Dieticians said it hindered information retrieval, explaining the relationship between diabetes and diet, and discussing treatment options. Furthermore, many dieticians found it more difficult to gather information on culturally influenced ideas about health and health‐care expectations and might not ask their patients about the questions they have.

D10: ‘Because of communication difficulties, it's difficult to ask in‐depth questions. It takes a lot of time. Then you're already happy if they eat 3 or 6 smaller meals instead of two large meals’.

D5: ‘It was just impossible. He didn't get me, and I didn't get him. Sometimes he recognised a word, and then understood it wrongly. (…) I would stop the conversation and arrange for an interpreter, I would just do that. It's no use, it's an hour of time wasted.’

Retrieving information about portion sizes was often mentioned as a challenge. One dietician mentioned:

D12: ‘They often can't read or write Dutch, so a food diary isn't an option then’.

All dieticians had experience with informal interpreters, like family members, and most had experience with professional interpreting services. However, none of the dieticians recently used these interpreting services, as they are no longer reimbursed.

Some dieticians were aware of the problems that may arise while using informal interpreters.

D10: ‘Definitely when they start talking amongst each other, you just can't follow it then. (…) Maybe they don't want to tell everything to the client. What happens there? You can't put a finger on that’.

The participating dieticians often did not know whether their patient understood the information. Only a few dieticians mentioned that it is important to check whether patients understand the information they were given.

D4: ‘And then, ‘do you have any questions? No, no questions, haha’. I wonder if they really get it. When I’ve explained and they go home’.

Because of the communication difficulties caused by a language barrier, explanation of the relationship between diabetes and nutrition is often avoided. This difficulty was not unique to migrant patients. It was also mentioned in relation to ethnic Dutch patients with a lower socio‐economic status, yet dieticians felt more restricted in their options to explain the relationship between diabetes and nutrition when a language barrier was present.

### 3.4 Skills: communication strategies and solutions

Dieticians felt that much creativity and effort is necessary for overcoming a language barrier. A few solutions to this were mentioned, such as asking the interpreter if they understood the information and to translate word‐for‐word. However, one dietician indicated she felt embarrassed to check the translation process. Other examples mentioned for facilitating communication were as follows: using simple language and visual materials to explain diseases, using photo books and household measures to estimate portion sizes, portraying things with their hands, and translating words via Google Translate. There were several dieticians who had information brochures in Turkish or Arab. However, one dietician mentioned that most materials with information on diabetes were not suitable.

D11: ‘I have been searching for materials with images (…) But I don't use most of them, because they've got too much text; too much, too small, or too difficult’.

One dietician explained that she was afraid of insulting her patients by using pictures during her consultation.

D1: ‘It's a bit of a hassle to work with all picture books, I really do not like that either. I find that, well, I think that comes across so childish’.

### 3.5 Knowledge: lack of knowledge about migrants’ background and culture

Dieticians perceived important cultural differences, for instance in food culture. They mentioned that many migrant patients used multiple carbohydrate sources per meal and more oils and butter than Dutch patients. Furthermore, they perceived that migrants more often had an irregular food intake pattern compared to Dutch patients. Most dieticians felt that they were not familiar enough with different food cultures and felt uncertain about differences in eating habits, preparation of dishes and therefore did not always know which probing questions are necessary during the food intake assessment. They wondered whether “fate” would play an important role in migrants’ ideas about disease aetiology.

D5: ‘Maybe faith has something to do with it. I believe that I once learned that at school. That they have learned from faith that the disease is their fate, like, they just have to endure it. Then I don't know to what extent it plays a role for that man’.

Consequently, many dieticians considered it difficult to obtain a complete overview of the food intake of their migrant patients, and therefore struggled with tailoring their advice to the patients’ habits.

D1: ‘You ask questions, but you don't know enough about particular habits to adequately respond to that. With Dutch people, often you know what they do and what they don't do, but with migrants this is more difficult’.

Dieticians dealt in different ways with incomplete information on the food intake of their patients. Some ask questions to check the information they gathered, like one dietician who asked about the amount of oil her patient bought per week, so she could estimate the intake per day. Some dieticians also made assumptions or used standard recommendations. For example, a few dieticians recommended replacing one of the two hot meals their patients used to eat by a bread meal, which is more commonly eaten by Dutch people.

D6: ‘I said that she should just replace the lunch by eating bread’.

However, some dieticians did feel confident about tailoring their advice to migrant patients. For example, the Bulgarian dietician explained:

D3: ‘I use the basics of what they eat and I build around that. When I went to a dietician myself, I couldn't eat soup in the morning anymore, I had to eat sandwiches. I don't do it that way. I look at what is achievable in their dietary pattern’.

Other perceived cultural differences dieticians felt insecure about were whether or not to shake hands and how to handle gender differences.

Most dieticians mentioned the social environment was important in migrant patients. Some of them said how family could be either supportive or a barrier for making diet changes. They generally asked about the family composition of their patients. However, only a few dieticians specifically mentioned that they asked their migrant patients about social support and pressure or difficulties of refusing foods in social situations.

### 3.6 Attitude: openness, respect and curiosity

Dieticians mentioned that having an open and respectful attitude towards all patients is important. They aimed at showing their patients they are interested to getting to know them and they are curious about their culture.

D4: ‘I think the most important thing is that you shouldn't think you know everything. And, well, to be very open towards what someone says and to ask questions. How they cook and how much they use of this or that. Or just take the time to have a conversation’.

A few dieticians mentioned ways to show their patients that they wanted to make an effort. For example, some dieticians mentioned that they would like to learn a few words in other languages and learn about frequently prepared traditional dishes.

D9: ‘Recognise certain words, mainly about foods, and show that’.

The Bulgarian dietician explained how she stimulates her patients to try to speak Dutch during a consultation. She realized how people can be insecure about their language skills and tried to help them overcome their insecurity:

D3: ‘Migrant people aren't open when communicating in the Dutch language because they are insecure. And if you show them you're also not that good at that, and you're also insecure, they appreciate that’.

Some dieticians were aware of their tendency to stereotype and wanted to avoid doing that.

D8: ‘In the Netherlands, we also don't wear wooden shoes and we don't eat “stamppot” (MJ: mashed dish) every day. You want to get away from the stereotyping a little’.

### 3.7 Attitude and skills: importance of building a trusting relationship

Trust was mentioned often as an important factor in the relationship between patient and dietician. Dieticians were aware that a trusting relationship facilitates the sharing of information.

D12: ‘Yes, creating that safe feeling that they feel like they can be themselves and that they can just communicate on an equal level. That you do not say “I am a caregiver,” but that you can talk to each other on an equal level and relax, so that they have the feeling that it is not a big barrier to go to you’.

Furthermore, small gestures that facilitated a warm interaction were mentioned as important for the relationship:

D11: ‘I always see it with my mom, she is 65 years old now and from the first generation and she likes it a lot when somebody treats her with warmth. Like her GP, who shook my mother's hand and cupped it with her other hand’.

However, some dieticians found it more difficult to build a trusting relationship with migrant patients, because of the language barrier and cultural differences.

D9: ‘Establishing a trusting relationship is more difficult because of language, because of culture, the piece of communication that's missing’.

### 3.8 Cultural competence training needs

None of the dieticians had received any training on cultural competence and many mentioned that they really wanted a course to be developed to this end. A few visited a mosque with a group of colleagues.

Dieticians would like to receive training that focuses on knowledge about culture in general, expectations of health care, eating habits, preparation methods and tools or images to explain the relationship between diabetes and nutrition in different languages.

D1: ‘Also what their entire culture is like and then I don't just mean what they eat, but also how they interact with each other. Because I can say, well I’ll make a list, I send it to you, and you have to do it. But, a woman would maybe accept that, but will a man do that? Like me telling him what to do. Do we shake each other's hand, or not. Do we do Ramadan, or not?’.

Some dieticians wanted to know a few words in their patient's language. A few also mentioned skills they wanted to learn, for example how to build a trusting relationship and how to convey information. Although language was mentioned as a major barrier during the consultation, only a few mentioned that they wanted to learn how to handle language differences.

The participants would prefer training methods that include role‐play as part of the communication training. Additionally, they would like migrant dieticians to share their best practices. Some dieticians would like to invite migrant patients as well, to ask about their experiences with a dietetic consultation.

D10: ‘But I’d also like to speak to people of that culture. How they experience a consultation with a dietician. More from their point of view. (...) So that you know what they need’.

## 4 DISCUSSION

This study reveals that dieticians struggle with their care for migrant patients and are not sure whether their care fulfils the migrant patients’ needs. Language differences were experienced as a major barrier hindering the necessary information retrieval and adjusting dietary advice to their needs. In particular, dieticians’ expectations on cultural differences in food habits complicated giving dietary advice. The language and communication barrier also hindered the building of a trusting relationship which they deemed essential for effective care. Dieticians expressed a need for training on culturally competent care, which they never received. Specifically, they would like to acquire cultural knowledge. In contrast, despite their experience of the importance of language barriers, many dieticians did not mention a need for communication training.

There is a large body of knowledge on the central role of language concordance in providing good quality of care. Language differences often lead to miscommunication, frustration and impede shared decision‐making.^15^ It also complicates talking about emotional matters^20^ and building a trusting and warm relationship, which is known to improve the quality and outcome of medical encounters.^21^ Although the use of trained professional interpreters positively affects patients' satisfaction, quality of care and health outcomes,^22^, ^23^ they are seldomly used; professional interpreter services are no longer reimbursed in the Netherlands. Alternatively, dieticians depend on informal interpreters, such as family members.

Although approximately 30% of the first‐generation migrant population in the Netherlands are known to be low literate,^24^ the participating dieticians showed little awareness of the impact of both low literacy and low health literacy. During the interviews, several situations were interpreted as non‐adherence or motivational problems, such as patients missing appointments, or that patients did not read the treatment plan, that in fact may be explained by limited literacy.^25^ These findings are in line with studies showing that health professionals have limited knowledge of health literacy, tend to overestimate patients’ health literacy 6, 26 and thus may misinterpret low health literacy as a lack of motivation. Furthermore, they do not know how to adequately support patients with low health literacy in diabetes self‐management.^6^, ^27^ In order to improve communication, health professionals should use clear language, dose their information, avoid medical jargon and use the teach back technique to allow the patient to teach back what they have heard.^28^

Besides literacy and health literacy, another theme that was not mentioned by the participants was the possibility of a lack of financial means of patients for buying healthy foods. In 2017, approximately 25% of non‐Western migrant households in the Netherlands had an income below the poverty line.^29^ As the higher price of healthy food is an important barrier for the consumption of a healthy diet,^30^ dieticians need to help their patients to achieve a healthy diet at a low cost.

It is well known that implicit bias, the projection of stereotypes onto individual patients in ways that affect clinical decision‐making,^31^ negatively influences health care.^32^ There were examples in the interviews that could reveal stereotyping. For example, one participant expected lower glycaemic control for her migrant patient. Furthermore, some dieticians expected migrant patients to be less motivated for behavioural change. On the other hand, some dieticians seemed aware that the problems they mentioned were not linked to the ethnic background of the patient. During the interviews, statements like “the same goes for Dutch patients” or “this does not apply to all patients from that ethnic background” were made regularly. Awareness of one's own implicit bias and prejudice can be taught, and this should be part of the cultural competence training.^31^

The experience of the respondents that food diaries were not returned, and food intake assessment was found difficult is not surprising, as written methods for food intake assessment, such as a food diary, are not suitable for people with low literacy. Verbal food intake assessment methods, such as a 24‐hour recall method or a dietary history method probably is a better fit for these patients. With respect to portion sizes, the use of visual aids is critical in migrant populations as the concept of individual servings may not exist for certain cultures, particularly those who eat from a communal serving dish. Food photographs or food models appear to be more effective than only using common household measures.^33^, ^34^

A previous study about the experiences of migrant patients with dietetic care showed that some patients were dissatisfied with their dietician because they expected a more rigorous and directive approach.^10^ This is in line with the experience of the dieticians in this study who felt that migrant patients often prefer a more directive approach and seem to be less interested in shared decision‐making. Although the concept of shared decision‐making may indeed be uncommon to migrant patients who are used to a more physician‐lead decision‐making health‐care system,^35^ recent studies in the Netherlands indicate that migrant patients also want to participate actively in consultations with their general practitioner. However, making an informed decision will be more difficult in case of a language barrier and low health literacy.^36^, ^37^ Motivational interviewing is designed to promote person‐centeredness and autonomy, and thus should always be tailored to the needs and skills of the patient involved.^38^ If health‐care professionals apply a strictly individualistic approach in their communication, this is likely to cause tensions in patients with a collectivistic cultural frame of reference.^39^, ^40^ Inclusion of family and significant others is important for effective engagement of all patients in shared decision‐making, particularly those from diverse cultural backgrounds.^35^, ^41^ Dieticians therefore should actively invite family members to accompany the patient during the consultation. Furthermore, to improve participation in shared decision‐making, patients may need more help and explanation from their dietician in order to understand the possibilities for diet modification. As a result, patients might be better equipped to share their preferred treatment.

It is impossible to have knowledge about all cultures because of hyper diversity and because the composition of the migrant and ethnic minority population is dynamic.^42^. Furthermore, it is debatable as to what kind of knowledge is relevant for culturally competent communication in dietetic care. Intra‐individual differences within cultures are large and focusing on having pre‐existing knowledge may reinforce stereotyping.^43^ Nevertheless, some knowledge aspects are important, as it helps dieticians to know which questions to ask. For example, knowledge that in some food cultures, rice may be prepared with large amounts of fats, which is uncommon in Dutch food culture, helps to improve skills to collect food intake information.

The feeling of uncertainty our respondents experienced due to the communication difficulties and lack of “cultural” knowledge was also observed in other studies in physicians and nurses.^44^, ^45^, ^46^ Training in cultural competences can diminish this uncertainty.^47^The participants in this study expressed a need for cultural competence training. Such a training should involve encouraging awareness of the social context of migrants in the Netherland, and how culture shapes individual behaviour and thinking in general. As communication difficulties were perceived as a major barrier to shaping a trusting relationship and to retrieve the necessary information, such training should have a strong focus on communication across language barriers and limited (health) literacy.

### 4.1 Strengths and limitations

To our knowledge, this is the first study to explore the experiences of dieticians caring for migrant patients with diabetes and the knowledge, attitude and skills they consider important for good quality dietetic care for these patients.

A limitation of this study was the small number of respondents with a migrant background; however, this reflects the fact that still few dieticians in the Netherlands are from ethnic minority background. It might be that more dieticians from different ethnic minority backgrounds would have elicited different experiences or communication styles during the interviews that would have diversified the data.

Data on the actual behaviour of dieticians during a consultation were not obtained. As dieticians may not be aware of all aspects of their own behaviour, these factors may have been missed. As direct observation of dieticians during consultation would provide objective data on culturally competent behaviours,^48^ this should be included in future studies in order to improve dietetic care.

### 4.2 Recommendations

Training in cultural competences should be developed and implemented for dieticians. Recommendations are provided in Table 2. The use of professional interpreters in dieticians consultations should be encouraged and reimbursed.

**Table 2:** Recommended knowledge, attitude and skills for training

Knowledge about cultural differences, including food habits an preparation of dishes
Knowledge and skills to educate patients to achieve a healthy diet at a low cost
Attitude of openness, respect and curiosity
Awareness of one's own cultural background and of implicit bias and stereotyping
Skills to communicate in case of a language barrier, including working effectively with informal interpreters
Skills to communicate with patients with low (health) literacy

## 5 CONCLUSIONS

Dieticians struggle with providing good quality dietetic care for migrant diabetes patients due to communication and language barriers, and difficulties in building a trusting relationship. They are conscious of their lack of “cultural” knowledge, and of the need of an open and respectful attitude, as well as communication skills for collecting and conveying information that are essential for culturally competent dietetic care. Dieticians seem unaware of the impact of low literacy and low health literacy in migrant patients. Cultural competence training could improve their attitude, knowledge and the skills needed to provide effective and acceptable dietetic care to migrants.

## CONFLICT OF INTEREST

None declared.

## Data Availability

The data that support the findings of this study are available on request from the corresponding author. The data are not publicly available due to privacy or ethical restrictions.
